# Intra-seasonal dynamics in metabolic processes of ^13^C/^12^C and ^18^O/^16^O in components of Scots pine twigs from southern Siberia interpreted with a conceptual framework based on the Carbon Metabolism Oscillatory Model

**DOI:** 10.1186/1471-2229-12-76

**Published:** 2012-05-30

**Authors:** Victor Voronin, Alexander A Ivlev, Vladimir Oskolkov, Tatjana Boettger

**Affiliations:** 1 Department of Bioindication, Siberian Institute of Plant Physiology and Biochemistry, Siberian Branch, Russian Academy of Sciences, 132 Lermontov Street, 664033, Irkutsk, Russia; 2 Department of Soil, Agrochemistry & Ecology, State Agrarian University, Timiryazevskaya Street 49, 127550, Moscow, Russia; 3 UFZ - Helmholtz Centre for Environmental Research, Department of Catchment Hydrology, Theodor-Lieser-Straße 4, D-06120, Halle (Saale), Germany

## Abstract

**Background:**

Carbon isotope data from conifer trees play an important role in research on the boreal forest carbon reservoir in the global carbon cycle. Carbon isotopes are routinely used to study interactions between the environment and tree growth. Moreover, carbon isotopes became an essential tool for the evaluation of carbon assimilation and transport from needles into reserve pools, as well as the allocation of stored assimilates within a tree. The successful application and interpretation of carbon isotopes rely on the coherence of isotopic fractionation modeling. This study employs a new Carbon Metabolism Oscillatory Model (CMOM) to interpret the experimental data sets on metabolic seasonal dynamics of ^13^C/^12^ C and ^18^O/^16^O ratios measured in twig components of Scots pine growing in southern Siberia (Russia).

**Results:**

The dynamics of carbon isotopic variables were studied in components of *Pinus sylvestris* L. in light and in dark chambers during the vegetation period from 14 June to 28 July 2006. At the beginning of this period water-soluble organic matter, mostly labile sugars (including sucrose as the main component) and newly formed bulk needle material, displayed relatively “light” δ^13^C values (depletion in ^13^ C). Then, ^13^ C content increased again with noticeable “depletion” events in the middle of the growth period. A gradual ^13^ C accumulation took place in the second half of the vegetation period. Similar effects were observed both in the light and in the dark with some temporal shifts. Environmental factors did not influence the δ^13^C values. A gradual ^12^C-depletion effect was noticed in needles of the previous year. The δ^13^C values of sucrose and proteins from needle biomass altered independently from each other in the light chamber. A distinct negative correlation between δ^13^C and δ^18^O values was revealed for all studied variables.

**Conclusions:**

The abrupt ^13^C depletion recorded by all tested trees for the period from June to July provides clear evidence of the transition from the dominant role of reserve carbohydrate pool (RCP) during the first half of the growth season to the preferable current year carbohydrate pool (CCP) consumption by new needles during its second half. The investigation of the isotopic signatures of *Pinus sylvestris* L. emphasizes the pivotal role of the intra-seasonal dynamics in carbon metabolism through the transport of assimilates from autotrophic (needles) to heterotrophic (twigs) organs of the studied trees. This provides an explanation for changes of carbon isotopic values observed within the growth season. The CMOM-based results support the hypothesis of the integration of three carbohydrate pools by photosynthesizing cells. The fluctuations of the carbon isotope ratios in different carbohydrate pools underlie various physiological processes in the tree metabolism. The possible mechanisms and pathways of formation of these carbohydrate pools are further discussed. Hence, CMOM provides a reasonable explanation for the absence of the impact of environmental conditions on the needle isotopic variables, the ^12^C-depletion effects and the use of RCP in needles. The model explains the negative connections between δ^13^C and δ^18^O values in all studied variables.

## Background

Research on the mechanisms of carbon isotope fractionation in conifers, their transport from needles into the reserve pools, as well as the dynamics of remobilization of the stored assimilates is important in the context of the role of the boreal forest as a C-storing reservoir in the global carbon cycle. The ^13^C isotope is frequently used as marker [1-3], to separate metabolites, and the natural ^13^C/^12^C ratios are used to understand the interactions between environment and trees [4-6]. Carbon isotopic signatures highly depend on cell metabolism, and their interpretations rely on the rationale of the correct isotopic fractionation modeling.

In the most recent carbon isotope studies the authors use the diurnal δ^13^C variations in tree respired CO_2_ to get information about possible pathways and pools of assimilates that could be the source of carbon for respiration. Gessler et al. [7] explain the short-term seasonal variations in δ^13^C sugars in the canopy of *P. sylvestris* by phloem loading and transport and uncouple the tree ring carbon isotope signal from C_i_/C_a_ ratio in the leaf. The ^13^C-labelling experiments [8] showed that functional groups in pyruvate differ in C allocation between respiratory pathways owing to different metabolic growth, maintenance and post-photosynthetic metabolism. In [9] a close correlation between the ^13^C abundance in day- and night-evolved CO_2_ was found. These authors concluded that there is a similar source of respiration carbon in the dark and in the light, while the metabolic pathways associated with CO_2_ production may change and thereby explained the different ^12^C/^13^C respiratory fractionations in the light and in the dark. Furthermore, it was found [10] that CO_2_ efflux in adult trees is supplied by recent photosynthates and carbon stores, but the contribution of these pools to growth and maintenance respiration remains unknown. The photosynthates in beech supplied the growth in early summer and refilled C stores during the late summer, while CO_2_ efflux in spruce was constantly supplied by a mixture of stored (with a dominant contribution) and recent photosynthates The temporal variation of post-photosynthetic fractionation related to changes in carbon allocation in different metabolic pathways is the most plausible mechanistic explanation for observed δ^13^C dynamics of respired CO_2_[11].

Nevertheless, all the authors admit the lack of a clear explanation of the origin of short-term δ^13^C variability in respired CO_2_ and organic carbon fractions on a diurnal basis. There is an urgent need for the use of supplementary modeling.

Farquhar and co-authors [12,13] showed in the 1980s that ^13^C-discrimination of photosynthesis is a function of the ratio between intercellular and ambient CO_2_ concentrations. The authors described diffusion and biochemical processes as main source of the isotopic discrimination. Their stationary balanced model explains the consistency of the physiological response of plants to changing environmental conditions mainly through the impact on plant stomatal conductance and net photosynthesis. The modeling of the relationship between carbon and water was particularly successful [4,5,13]. However, this fixed-state approach yields insufficient insight necessary for the interpretation of short-term or intramolecular carbon isotopic fractionation.

To fill this gap, a new approach grounded in the idea of oscillating metabolic processes was developed [14,15]. The discovery of substrate pool fluctuations in a cell (filling/depletion) was made during studies of isotopic ratios of different photosynthesizing organisms. These studies described the Rayleigh effect in cell processes suggesting repeated depletion of carbon substrate pools [15,16]. Photosynthesis was considered as a short-term two-phase oscillation: CO_2_ assimilation and photorespiration. The photosynthetic oscillations are regulated by the key enzyme – RuBisCO – that can be both carboxylase and oxygenase. Shifting between these two enzyme functions depends on the CO_2_/O_2_ concentration ratio, which fluctuates with time. As a result, CO_2_ assimilation corresponds to the carboxylase phase of RuBisCO, while photorespiration belongs to the oxygenase phase.

**Figure 1:**
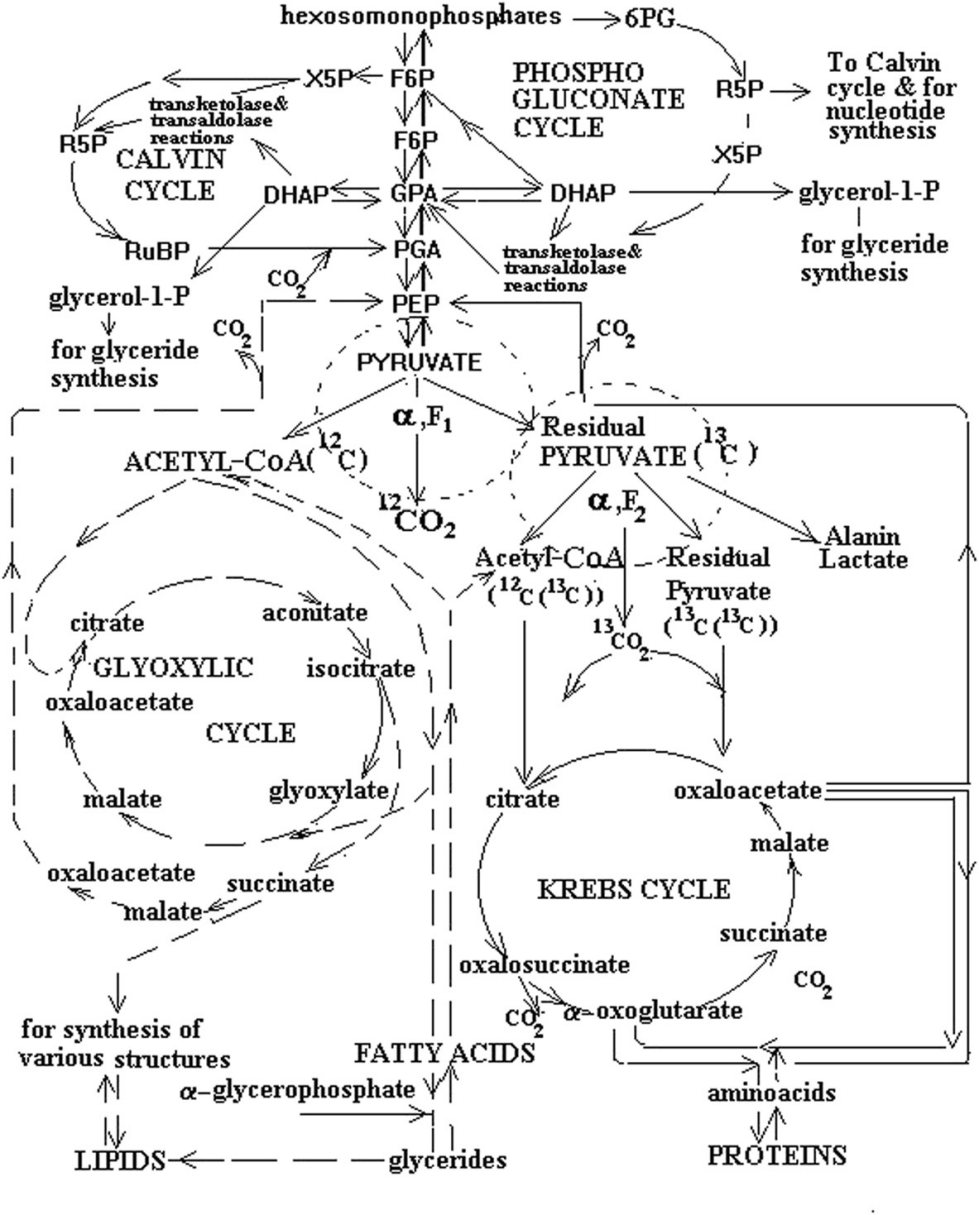
**Simplified diagram of the glycolytic metabolism.** According to the OMCM, the glycolytic chain functions in two regimes – the phase of glycolysis and the phase of gluconeogenesis. During glycolysis, the carbon substrate flux goes “down”. This corresponds to the transformation of carbohydrates into lipids and proteins. During gluconeogenesis, the carbon substrate flux goes “up”. This corresponds to the reverse transformation of lipids and proteins into carbohydrates. “Up” and “down” indicate only general direction of transformations, since glycolytic and gluconeogenetic pathways do not coincide. There are two stages of the pyruvate decarboxylase complex in glycolysis: one corresponds to less than twofold pyruvate pool depletion and mainly comprises lipid component synthesis via C_2_-fragments depleted in ^13^C relative to carbohydrates, which feed the glycolytic chain. The other corresponds to more than twofold pool depletion and comprises the Krebs cycle operation and the derivation of the protein components. The products of pyruvate decarboxylation used as structural units in the Krebs cycle operation (C_2_ and C_3_- fragments and evolved CO_2_) are enriched in ^13^C. According to the known Rayleigh equation [15], isotope ratios of initial substrate, reaction product and residual substrate in chemical reactions are linked in the following way: when the extent of the initial substrate pool is depleted less than twofold, the product is enriched in ^12^C with respect to the initial substrate; when the extent of depletion is more than twofold, the product gets enriched in ^13^C. Dotted lines denote the enzymatic pyruvate decarboxylase complex where carbon isotope fractionation occurs. Abbreviations: X5P, xylose-5-phosphate; R5P, ribose-5-phophate; RuBP, ribulose-1,5-bisphosphate; 6PG, 6-phosphogluconate; F6P, fructose 6-phosphate; FBP, fructose 1,5-bisphosphate; PGA, phosphoglyceric acid; DHAP, dihydroxyacetone phosphate; PEP, phosphoenolpyruvate

The secondary metabolism is considered as a long-term and also two- phase oscillations of glycolysis and gluconeogenesis (Figure 1). Carbon flux in the glycolytic chain goes “down” in the glycolysis and “up” in the gluconeogenesis. The oscillations in the glycolytic chain are regulated with the fructose-1.6-bisphosphate futile cycle, which periodically alters the allocation of carbon substrate according to the energy needs of the metabolism [17,18]. The glycolysis phase of oscillation corresponds with the dark period, while the gluconeogenesis phase corresponds with the light period. The combination of these two concepts provides the basis for the Carbon Metabolism Oscillatory Model (CMOM) [15].

According to the CMOM, carbon isotope fractionation occurs in both phases of photosynthesis: CO_2_ assimilation and photorespiration. Isotope effects during CO_2_ assimilation are explained by RuBP carboxylation during the carboxylase phase. This results in ^13^C depletion of assimilates and practically uniform distribution of carbon isotopes within the Calvin cycle. This conveys the ^13^C depletion in plant biomass relative to assimilated CO_2_.

The carbon isotope effect in photorespiration is associated with glycine decarboxylation during the oxygenase phase of active RuBisCO. Photorespiratory products and labile carbohydrates synthesized in this phase are greatly enriched in ^13^C relative to the substrate glucose-6-phosphate (G6P) resulting from the carboxylase phase. Due to the kinetic nature of the carbon isotope effect in glycine decarboxylation, intramolecular patterns of carbohydrates and photorespiratory products are characterized by unbalanced carbon isotope distribution [19]. Contrary to the carbon isotope effect in CO_2_ assimilation, photorespiration reduces ^13^C depletion in the biomass. Thus, the effect has opposite signs for CO_2_ assimilation and photorespiration.

In the glycolytic chain, carbon isotope fractionation occurs in the glycolysis phase and relates to pyruvate decarboxylation (Figure 1). This reaction takes place at the crossing point of the central metabolic pathways. Their products are the main structural units used during post- photosynthetic metabolism. Because of the timing and kinetic nature of the isotope effect, lipids become ^13^C depleted relative to proteins. This also provides heterogeneity of their isotopic components. Additionally, the Rayleigh isotope effect accompanying the pyruvate pool depletion contributes to further variations in isotopic signatures of individual components synthesized at that time (different levels of pool depletion) [20].

There are a few studies supporting the CMOM concept. Roussel et al. [21] experimentally demonstrated the presence of oscillations in the metabolic pathways of photosynthesis. They analyzed CO_2_ concentration near the compensation point of the subcellular space of tobacco leaves by using a fast-response CO_2_ gas exchange system. Their observations indicated that CO_2_ concentration might fluctuate in a matter of a few seconds because of the reverse relationship between CO_2_ assimilation and photorespiration.

Igamberdiev et al. [22,23] confirmed the opposite sign of the carbon isotopic effect in CO_2_ assimilation and photorespiration by comparing δ^13^C of leaf biomass of GDC-deficient mutants (artificially grown plants with genetically modified glycine decarboxylase complex, GDC) and wild species of selected plants. Ivlev [24] showed that the observed intramolecular patterns of certain plant elements and isotopic differences of metabolites isolated from plant biomass imply assimilatory and photorespiratory carbon fluxes in plant cells.

Recently Dubinsky and Ivlev [25] revealed the most probable cause of the persistent photosynthetic oscillations. A simple photosynthesis model for measured cell parameters demonstrated counter-phase oscillations in CO_2_ and O_2_ concentrations controlled by RuBisCO. The duration of the oscillations was 1–3 seconds. Roussel and Igamberdiev [26] described different mechanisms of metabolic regulation based on nonlinear dynamics and showed that the photosynthetic oscillations may result from the competition between two substrates (CO_2_ and O_2_) and the delay in CO_2_ release following the oxygenation of RuBP in photorespiration.

There is a clear difference between explanations of metabolic isotope effects using the CMOM and steady-state models. For example, a steady-state model estimates a constant carbon isotope ratio for dark-respired CO_2_. Thus, the effect of carbon isotope fractionation does not change. According to the oscillatory model, the δ^13^C value of CO_2_ during dark respiration changes depending on many factors including two isotopically different carbohydrate pools developing during different phases of oscillations. These pools are the sources of dark-respiration substrates whose contribution depends on the pool size and dark-respiration duration, which are related to the physiology of the plant species and environment.

The Rayleigh effect is another reason that δ^13^C fluctuations in the dark-respired CO_2_ need to be studied further. The oscillatory model describes δ^13^C values of dark-respired CO_2_ with a dynamic variable. Perhaps the carbon isotope ratio of the evolved CO_2_ depends on the pyruvate pool depletion of the glycolysis and the Krebs cycle during continuous oscillations in the glycolytic chain.

The steady-state model fails to interpret irregularities in enrichment of ^13^C in CO_2_ during “light enhanced” dark-respiration (LEDR) [27]. The CMOM explains this as a result of photosynthetic oscillations and the isotope effect of photorespiration. Moreover, labile carbohydrates accumulated in the oxygenase phase include glucose components with irregular carbon isotope distribution. Atoms C-1, C-2 and C-5, C-6 of glucose (two C_2_-fragments) synthesize organic acids during dark respiration [27] whereas most of ^13^C-enriched C-3 and C-4 atoms of the residual glucose are used to form LEDR CO_2_. This is confirmed by the fact that the ^13^C enrichment of LEDR CO_2_ strongly depends on light intensity [28] as well.

The two models rationalize the isotopic fractionation in plant metabolites in different ways. The steady-state model links the isotope fractionation of various biochemical reactions to appearance of the branching points throughout the metabolic reaction network. All isotopic effects are interconnected with each other within the same carbon flow and appear concurrently along the chemical chain. Thus, the isotopic compositions of metabolites are very difficult to predict [29,30]. The CMOM explains the isotopic fractionation by means of specific metabolic pathways and the Rayleigh effect.

The intriguing problem of tree metabolism theory regarding the striking ^13^C enrichment of heterotrophic organs (seeds, stem, twigs, etc.) relative to autotrophic organs (leaves and needles) is assessed differently by these two models. The steady-state model provides no clear answer because of its assumption of temporal coincidence and continuity of all processes in a cell. It can explain only some aspects of that problem, for example, variations in biochemical components between leaves and heterotrophic organs, or seasonal variations in δ^13^C values of leaves and heterotrophic tissues, or daily variations of growth substrate (sucrose), etc. [31].

The oscillatory model proposes a coherent explanation of the relative ^13^C enrichment of heterotrophic organs by linking its origin to labile carbohydrates. The CMOM recognizes labile carbohydrates as the main carbon source for heterotrophic growth [32]. On the other hand, labile carbohydrates are enriched with ^13^C (like other photorespiratory products) since they were synthesized during the oxygenase phase of photosynthetic oscillations. Gessler *et al*. [33] provided experimental evidence of this conclusion. A considerable part of the water-soluble fraction of leaf organic matter contains labile carbohydrates enriched with ^13^C unlike those of the insoluble fraction. This conclusion corresponds well with the CMOM model since the insoluble fraction includes mainly proteins and lipids synthesized with the starch pool of the carboxylase phase. Likewise, this model explains the same range of δ^13^C variations in the water-soluble organic matter of leaves and phloem sap. The former is the main source for the latter.

This study aims to understand the role of metabolic processes in substrate transport and storage pool formation in pine trees growing in a boreal forest of southern Siberia. Our analysis focuses on examining the carbon and oxygen isotopic signatures in needles and twig components of *Pinus sylvestris* L. placed in light and dark chambers during the growth season. The CMOM was applied for interpretations of the results. Mechanisms of carbon isotope fractionation during the metabolism are studied in great detail, whereas the oxygen isotope fractionation was used only to demonstrate the impact of photorespiration on biomass oxygen isotope components coupled with carbon isotopes during CO_2_ assimilation.

## Results and discussion

**Figure 2:**
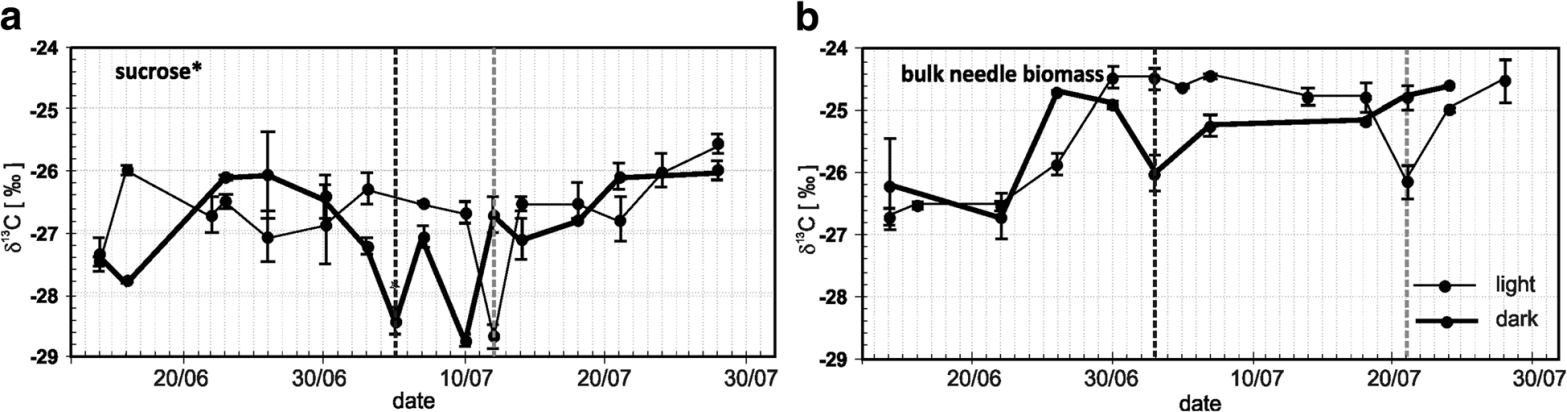
**δ**^**13**^C values of sucrose* (a) and bulk needle material (b) of 2-year-old pine branches, placed in light (thin lines) and dark (thick lines) chambers during the vegetation period in summer 2006. Stars mark the sharp peaks on the δ^13^C curves. Dotted lines mark the halves of growing period in the light (gray) and dark chambers (black). *Water soluble organic fraction consisted of ≥97 % sucrose. Data is shown as mean values ± SD (n ≥ 3)

Figure 2a-b shows variations of δ^13^C of bulk needle material and sucrose (water soluble organic fraction consisted of ≥97% sucrose) synthesized in both light and dark conditions from 14 June 2006 to 28 July 2006. This period includes (i) the phase of active growth (the first half of the vegetation period) in the twigs and new needles until the beginning of July and (ii) the phase of final growth (the second half of the vegetation period). The growing season in this region is less than 60 days.

The measured δ^13^C of both sucrose and biomass of needles fluctuates in the light and dark chambers throughout the observed period. Both components display different temporal shifts of the observed peaks in the dark and light conditions (Figure 2a,b). Sucrose is the main form of the transported substrates and a carbon resource for the metabolite synthesis [e.g. [32]. Therefore, the δ^13^C course of the biomass generally follows the δ^13^C level of sucrose but is ^13^C-enriched compared to sucrose. Considering sucrose as the main source of carbon for biomass synthesis, which is accompanied by isotope effects of the pyruvate decarboxylation, we can conclude that the carbon isotope composition of the biomass should differ from that of sucrose to some degree due to respiration.

The CMOM suggests three isotopically different carbohydrate pools formed by photosynthesizing cells in needles with various pathways and relevant for different time intervals according to the respective metabolic needs. As a result, the complete mixing of carbon flows does not occur in the process of the metabolism and the initial differences of δ^13^C values of carbohydrates from the individual pools remain.

Analyzing the isotopic changes in CO_2_ respired by the tree leaves in the light and in the dark Tcherkez et al. [9] supposed that the reasons for the changes might be the difference in metabolic pathways which may change with time, but not the pools having different carbon isotope ratios as follows from CMOM.

De Wit et al. [34,35] suggested that a carbohydrate pool develops during photosynthesis, which is used by needles for respiration and for tree growth throughout the vegetation period. Its dynamics are regulated by the concentration of labile carbohydrates such as glucose, sucrose and other assimilates (raffinose, galactose, etc.) in various organs of a tree [36]. We link this putative pool of carbohydrates to carbohydrates formed in the gluconeogenesis phase via the glycolytic oscillations. This CMOM assumption explains relatively “light” (similar to lipids) isotopic signatures of fresh bulk needle material and sucrose in the initial phase of active growth. It is likely the RCP is developed during the final phase of the preceding vegetation period, once the growth is reduced or terminated and the tree is transitioning to winter dormancy and the onset of the next vegetation period.

The sharp peaks on the δ^13^C curves of sucrose (Figure 2a) can be explained by a change in response of isotopically different carbohydrate flows over time. At night, when the Calvin cycle is not activated, the “heavy” photorespiratory carbohydrate flow from old needle cells ceases, whereas the ^13^C-depleted “light” flow from the reserve photosynthetic carbohydrate pool (RCP) continues. The existence of different carbohydrate pools as well as the pool of recent photosynthates was assumed in [10], but it was admitted that the contribution of these pools to growth and maintenance remains unknown. Furthermore, it was suggested that these C stores are formed at different stages of the growth period.

Figure 2a and 2b show distinct ^13^C depletion events for the needle sucrose on July 5^th^ and 10^th^ in the light and July 12^th^ in the dark chambers. The ^13^C depletion in bulk needle material was documented on July 3^rd^ in the dark chamber and July 21^st^ in the light chamber. These events tend to take place around the middle of the growth period and probably indicate the transition from the first active phase of the vegetation period to the second one. The reason for the splitting of ^13^C depletion of sucrose into two peaks in the dark chamber (Figure 2a) is not yet clear.

The nature of the ^13^C depletion in both needle sucrose and biomass (as growing parts of the trees) cannot be explained by the influence of environmental factors alone. It is unlikely that ^13^C depletion signatures are highly correlated with observations of light intensity, precipitation, soil moisture and temperature. For example, a 3-day rainstorm (9 July 07 – 11 July 07) did not affect the carbon isotopic signatures. All together, the effect of ^13^C-depleted RCP and the variable carbohydrate flow probably mask the influence of immediate variations in temperature, light etc. The “mask” effect was noticed in [7], but the authors related it to phloem loading and transport processes.

**Figure 3:**
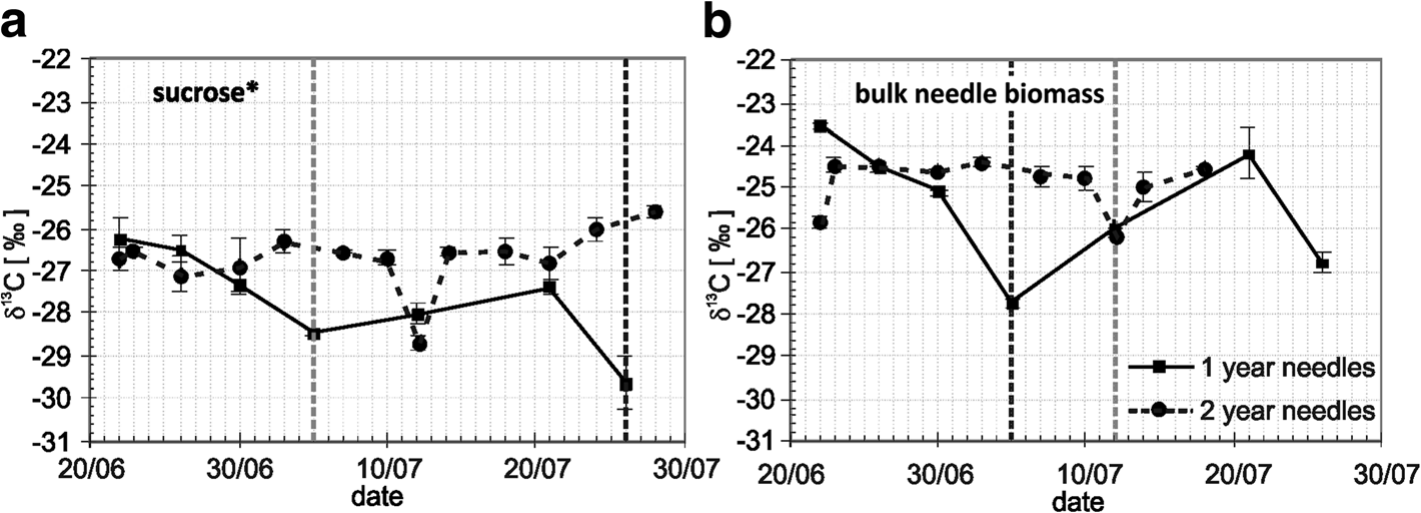
**Courses of δ**^**13**^C values of sucrose* (a) and bulk needle material (b) in needles of first (continuous black lines) and second (dotted black lines) year for the vegetation period 2006. Dotted vertical lines mark the halves of growing period for the 1-year-old (black) and 2-year-old needles (gray). *Water soluble organic fraction consisted of ≥97 % sucrose. Data is shown as mean values ± SD (n ≥ 3)

Clearly, the variations in the use of the RCP cause the fluctuations in carbon isotope signatures of biomass components of the studied pines and mask the short-term relationship between the δ^13^C values and environmental conditions. We assume that the ^13^C depletion probably works like an ontogenetic clock. When the newly formed needle cells start their own photosynthetic assimilation, the assimilate flow associated with the Calvin cycle is insufficient to feed the glycolytic chain with substrates, and the RCP compensates this deficit. The flow of “light” substrates originating from the RCP leads to a further ^13^C depletion while passing through the pyruvate dehydrogenase complex, and develops extremely “light” carbohydrates that may cause the abrupt depletion events (Figure 2a). Therefore, these phenomena can be linked to the transition from the phase of active growth to the phase of assimilate accumulation. These effects were registered under various environmental conditions in all our experimental data of bulk needle material and its sucrose from the dark and the light treatments (Figures 2a and 2b), and δ^13^C of twig cellulose in the current and previous vegetation seasons (Figure 3a). This supports the tentative hypothesis of ontogenetic origin of the ^13^C-depleted carbohydrates, which needs to be additionally verified.

Furthermore, the common pattern of the δ^13^C signatures among all studied (Figure 2a and 2b) plots includes: (i) sawtooth-like variations of δ^13^C components and (ii) abrupt decrease in δ^13^C values during the transition from the first half of vegetation period to the second half. The pattern also includes various offsets of negative sucrose δ^13^C peaks and the time intervals between corresponding peaks of bulk needle material. The peaks in sucrose appear much closer to each other than those in bulk needle material, which is hard to explain by means of the CMOM.

Figure 3 shows δ^13^C signatures of sucrose and biomass of the year 2006 and year 2005 in needles sampled from the light chambers. The sucrose ^13^C of the first-year needles is substantially ($\hat{t}$ = 1.97; Φ = 13; α = 0.05) depleted in comparison to sucrose ^13^C of the second-year needles. Student’s t-test ($\hat{t}$= 0.58; Φ = 21) shows insignificant differences in the mean of the needle biomass δ^13^C of the first year and the second year (Figure 3b).

Metabolism coupled with the process of respiration results in gradual scavenging of ^12^C isotopes in needles, since the RCP does not contribute significantly to the needle formation in the following year. Similar processes trigger the isotopic imbalance in needle and biomass carbohydrates as well. However, the ^12^C-scavenging effect in the case of biomass is weaker. The result agrees with conclusions of other studies e.g. [37,38], which suggest the remobilization of carbohydrate reserves stored in the prior year occurs predominantly during the process of wood formation at the beginning of the vegetation period.

Newly formed RCPs may impact the carbon isotope composition of tree-ring cellulose components for several years. Boettger and Friedrich [39] found a one-year memory effect in δ^13^C-time series of *Pinus sylvestris* L. growing at the northern tree line of the Khibiny Mountains (NW Russia) and firs (*Abies alba* Mill.) growing in Franconia (central Germany). Moreover, individual trees show even longer memory effects lasting for two and four years in pine and fir, respectively [39]. The deferral pattern in the RCP depletion was explained by: i) limitations in the availability of the reserve substances in the trees growing at their distribution limit in contrast to the trees growing in favorable conditions, and ii) differences in storage of the carbohydrates by different tree species [40].

**Figure 4:**
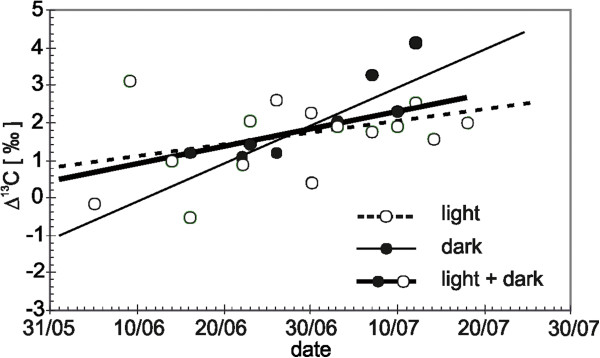
**Differences (Δ) between δ**^**13**^C values of sucrose* and δ^**13**^**C of biomass from needles measured in the light and in the dark during the vegetation period 2006.** *Water soluble organic fraction consisted of ≥97 % sucrose

The results shown in Figures 3a and 3b indicate that during the first half of the vegetation period the bulk needle material is slightly enriched with ^13^C, in contrast with needle sucrose. The enrichment occurs during the period of heterotrophic growth due to the isotope effect of pyruvate decarboxylation in the glycolytic chain due to respiration releasing ^13^C-depleted CO_2_ (Figure 4). In the second half of the vegetation period, ^13^C enrichment of sucrose increases relative to ^13^C biomass because the newly formed cells begin photosynthesis and the influx of carbohydrates from the CCP increases significantly due to photorespiration.

This effect is less pronounced in the dark chambers as compared to the light chambers. The slopes of differences between δ^13^C values of sucrose and biomass of needles sampled in the dark and in the light conditions (Δ^13^C) are 0.03 and 0.10, respectively (Figure 4). The gradient in the dark chamber values is small because the Calvin cycle does not operate in the darkness, and the “heavy” substrates formed in the oxygenase phase probably do not contribute significantly to the biomass formation. Thus, the δ^13^C variations in sucrose and biomass of the first and second year needles confirm once again the fluctuations of isotopic signatures of sucrose and biomass over time.

**Figure 5:**
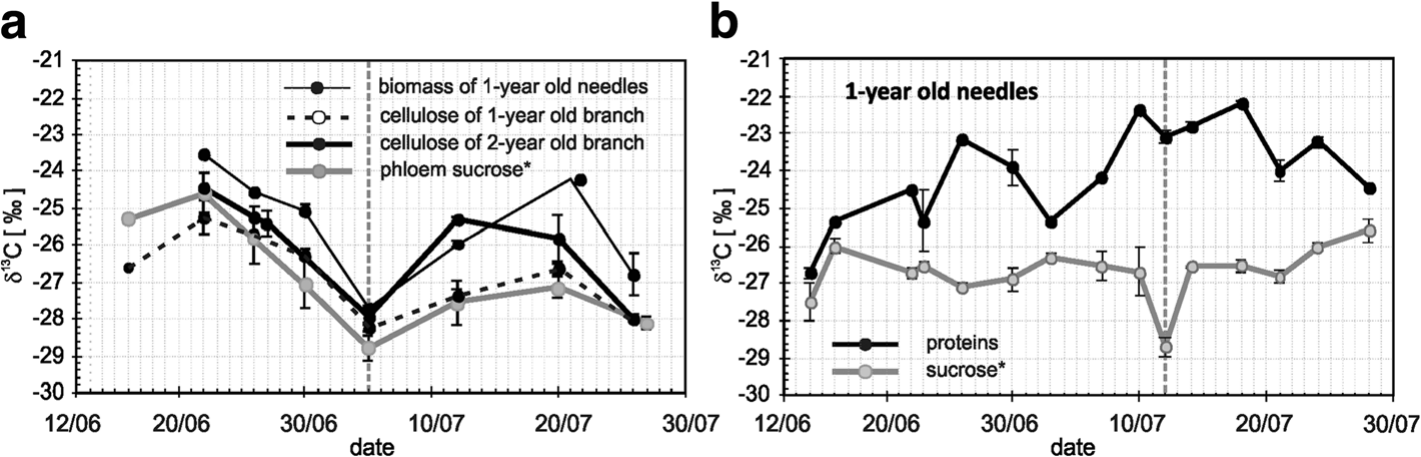
**a) δ**^**13**^C course of 1-year old bulk needle material (thin line), cellulose of 1-year-old (dotted black line) and 2–year-old (thick black line) twigs and phloem sucrose* (thick gray line) in the light during the vegetation period 2006; b) δ^**13**^**C course of needle sucrose (thick gray line) and proteins (thick black line) in the light chamber during the vegetation period 2006.** Dotted gray lines mark the halves of growing period. *Water soluble organic fraction consisted of ≥97 % sucrose. Data is shown as mean values ± SD (n ≥ 3)

Figure 5a shows clear evidence of ^13^C depletion for sucrose and bulk needle material in the middle of the vegetation period. The correlation of δ^13^C trends indicates a close relationship between all studied components, which confirms the regular replacement of labile carbohydrates from the RCP and the CCP in the first half and the second half of the vegetation period, respectively.

The δ^13^C trends of sucrose and proteins isolated from the biomass of needles grown in the light chamber (Figure 5b) show significant ^13^C enrichment in proteins. This is a result of carbon isotope fractionation in pyruvate decarboxylation corresponding to the Rayleigh effect and isotopic balance. Sucrose is the initial substrate feeding the glycolytic chain, whereas proteins are the product of their next transformation including those occurring in the pyruvate dehydrogenase complex (Figure 1). The role of pyruvate in C allocation between respiratory pathways, which may be different in growth, maintenance and post-photosynthetic metabolism, was underlined in [8]. This is in line with the CMOM’s assertion of a different role of the reaction in carbon isotope distribution at different functional states of the organism.

The carbon isotopic composition of sucrose and proteins depends on various factors. The sucrose δ^13^C depends mainly on the isotope effects during CO_2_ assimilation and photorespiration, while the protein δ^13^C varies depending on the isotope effects of the pyruvate decarboxylation and the lipid-carbohydrate and protein-carbohydrate metabolism. Likewise, oxygen resulting from water reduction during CO_2_ assimilation is another important player in the processes of photosynthesis and photorespiration. In order to demonstrate the interactive relationship between oxygen and carbon composition, fluctuations in the oxygen and carbon isotope ratios from pine biomass components were compared.

**Figure 6:**
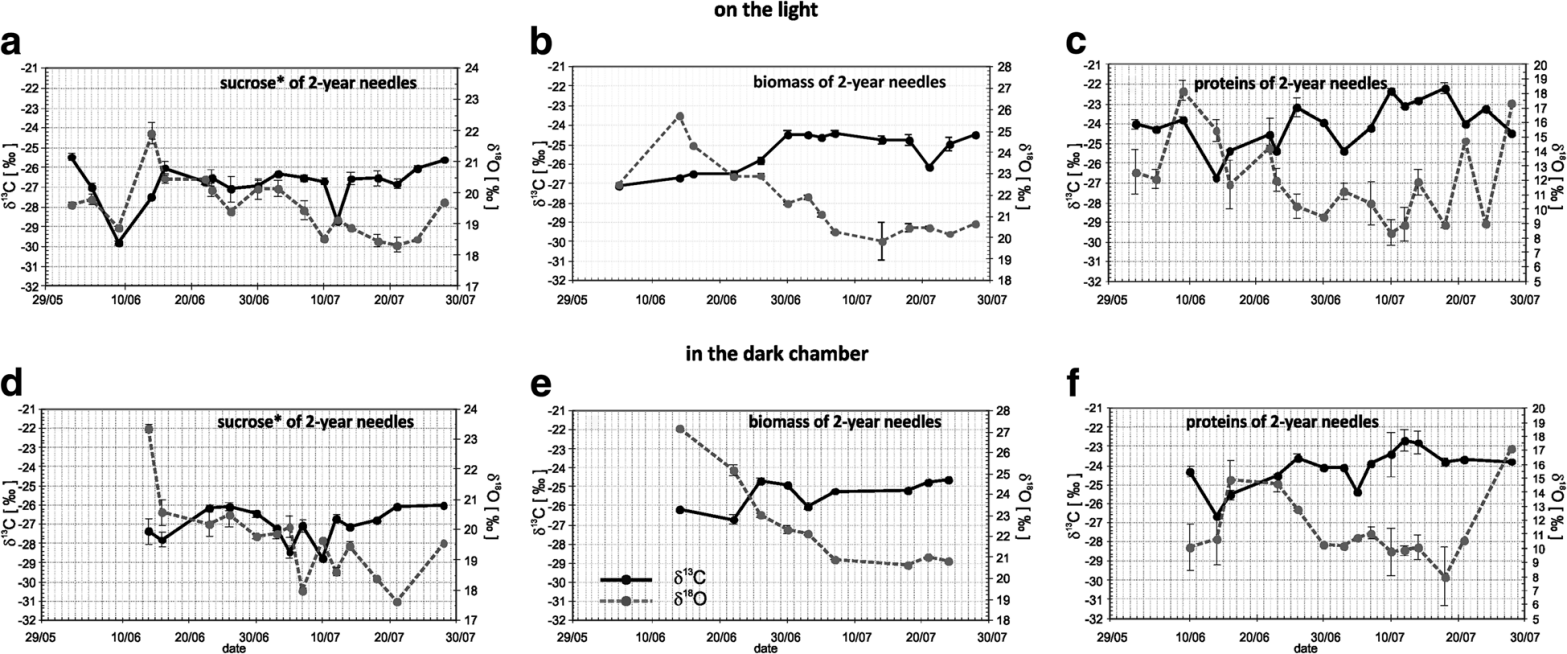
**The courses of δ**^**13**^**C (black lines) and δ**^**18**^O (dotted gray lines) of sucrose* (a,d), biomass (b,e) and proteins (c,f) from the light (upper panel) and dark (lower panel) chambers during the growth season 2006. *Water soluble organic fraction consisted of ≥97 % sucrose. Data is shown as mean values ± SD (n ≥ 3)

Figure 6 clearly shows a negative correlation between the δ^13^C and δ^18^O values of all corresponding variables. The correlation coefficients between δ^13^C and δ^18^O values of biomass and proteins from the light chamber and δ^13^C and δ^18^O values of biomass from the dark chamber are significant and equal to −0.70 (n = 14) at p < 0.01, -0.50 (n = 18) at p < 0.05 and −0.71 (n = 9) at p < 0.05, respectively. Interestingly, the corresponding isotope ratios of carbon and oxygen in the stem cellulose are significantly correlated (r = −0.86 at p < 0.01, n = 11) as well (not shown in Figure 6). However, the correlation between carbon and oxygen isotope signatures in the sucrose is insignificant (Figure 6a and 6d) for both the light and dark chambers, and proteins synthesized in the dark (Figure 6d).

Clearly, the isotopic characteristics of CO_2_ and O_2_ are strongly correlated since these gases participate at the same stages of the photosynthesis process. It is well known that the isotope composition of oxygen in photosynthesis corresponds to the rate of oxygen consumption in photorespiration e.g. [41,42]. The fractionation of oxygen isotopes results in the preferred involvement of ^16^O in the process of photorespiration and, partially, in the process of biosynthesis, whereas the “heavy” isotope ^18^O is accumulated in O_2_[43] and released by the cells into the atmosphere [44].

The negative relationship between δ^13^C and δ^18^O values (Figure 6) is possibly caused by the Rayleigh effect on the CO_2_-assimilated carbon isotope and the oxygen isotope absorbed during photorespiration due to photosynthetic oscillations. The rate of ^18^O-enrichment in molecular oxygen depends on the degree of O_2_ involvement in photorespiration, which differs from the amount of O_2_ generated through the photosynthetic reduction of CO_2_. The amount of produced O_2_ is determined by the proportion of the amount of reduction of CO_2_ diffused into the cell and CO_2_ available for assimilation. This means that as more CO_2_ is involved in assimilation and as the assimilated carbon (biomass) becomes more ^13^C-enriched, more oxygen will be produced.

Furthermore, the greater the proportion of produced oxygen, the more “lighter” oxygen (^16^O-enriched) is included in the process of photorespiration and biomass synthesis (Figure 6b and 6e).

The δ^13^C and δ^18^O values of bulk needle material, needle sucrose and needle protein are negatively correlated (Figure 6b, 6a and 6c). However, the relationships between the δ^13^C and δ^18^O values of biomass components are not strong, since most of the oxygen in the biomass oxygen originates from CO_2_. The results suggest a reciprocal interaction between the Rayleigh effects during CO_2_ assimilation and photorespiration.

## Conclusions

1. Fluctuation of the reserve carbohydrate pool (RCP) is a key factor in the dynamics of carbon isotope signatures of all biomass components (sucrose, proteins and cellulose) from needles, twigs and stems of studied trees (*Pinus sylvestris* L.) recorded during the vegetation period. The carbon isotope signatures relate to the ^13^C-depleted isotope pool formed mainly through glucose remobilized during the gluconeogenetic phase of the glycolytic chain oscillations. The RCP consumption dynamics during the growth season mask the short-term relationships between the δ^13^C values and current environmental conditions.

2. The analysis of both sucrose and biomass of needles and twigs for the δ^13^C signatures using the Carbon Metabolism Oscillation Model (CMOM) suggests involvement of three different carbohydrate pools (depending on the ratio of carbon isotopes) in photosynthesizing cells. The isotopic compositions of studied pine trees emphasize the importance of timing in the transport of assimilates from autotrophic to heterotrophic organs. The dynamics of carbon transfer appear to change throughout the vegetation period.

3. Transition from the first to the second half of the vegetation period correlated with major depletion of ^13^C among all analyzed needle and wood components, which most likely corresponds to the time when the source of carbon substrates in growing needles changes from the RCP to freshly synthesized assimilates (CCP). The magnitude and rate of ^13^C depletion are probably regulated by both the specifics of metabolic processes in pine trees and environmental conditions of the previous and current years.

4. The significant negative correlation between δ^13^C and δ^18^O values of bulk needle material and its protein fraction from the light chambers, and biomass from the dark chambers is the result of coupling the CO_2_ assimilation and photorespiration processes. Fractionation of carbon and oxygen isotopes occurs in both these processes. The data define the relationship between the Rayleigh effects during CO_2_ assimilation and photorespiration and suggest similar mechanisms of isotope fractionation for carbon and oxygen.

5. The Carbon Metabolism Oscillatory Model provides a good explanation of the dynamics of the isotopic signatures. The CMOM helps to understand (i) the transport of assimilates, and (ii) the mechanisms related to formation of the metabolic pools using isotopes.

6. The mechanism of carbon isotope fractionation in a cell described above is common to any photosynthesizing organism. The existence of isotopically different carbon pools makes it possible to investigate the temporal organization of assimilate transport and can be assumed for any conifer. Mechanism and pathways of photosynthate transport, reserve pool formation and temporal pool remobilization depend on environmental conditions and are species-specific.

### Climate and geography of the study area

The climate of the studied region is extremely continental with a high solar irradiation in the summer. The frost-free period continues for about 100 days. Light frosts (from −1°C to −4°C) occur occasionally during the growth season, for example, in late spring (before June 15) and early autumn (after August 25). The annual temperature amplitude is up to 80°C [45], and the daily temperature variations may exceed 20°C. January mean temperature is as low as −23.4°C and the mean temperature for July is only 17.2°C. The annual average temperature is −1.6°C, and precipitation is 359 mm. An annual precipitation maximum occurs during the second half of summer. The snow cover is about 40–50 cm deep. Evaporation exceeds rainfall during the warm season.

The study plot is situated on a gentle eastern slope (2-3°). The type of soil is gray forest non-podzolized loamy soil on Jurassic rock underlain by sands. The depth of the groundwater table is between 11 and 50 m.

## Materials and methods

The field experiment was done on a plantation of conifer seedlings at the research field station of the Siberian Institute of Plant Physiology and Biochemistry SB RAS located on the outskirts of Irkutsk City, Russia (52^0^14' N, 104^0^16' E).

### Sampling

The needle samples, wood and phloem of twigs of *Pinus sylvestris* were collected in summer 2006. Three pine trees from comparable habitats were selected for the experiment. Cylinder-shaped assimilation chambers coated with polyethylene and reinforced with a lightweight firm frame were mounted in the middle part of the tree crowns. The twigs from the transparent chambers were exposed to daylight. The “dark” chambers were covered with black fabric and aluminum foil to prevent overheating inside the chamber. The airflow through the chambers was about 40 liters per minute. The temperature inside the chambers and the ambient air temperatures were measured with copper constantan thermocouple sensors equipped with a multi-point data logger KSM-4 (Russia).

According to previous experience, the maximum rate of pine photosynthesis was observed from 9.00 to 10.00 a.m. The depression of photosynthesis starts at about 11.00 a.m. due to high air temperature [46]. Therefore, the sampling of needles, wood and phloem from twigs in both chambers was carried out once a day at 10:00 a.m. This was preceded by the installation of the chambers on the twigs at 9 a.m. for 1 hour. The dark chambers were kept in the dark for 1 hour.

### Sample preparation

A part of the needle samples was dried and ground in a rotor mill for isotope measurements on the bulk needle material. The rest of the sample material was used for the extraction of sucrose and proteins for further isotopic measurements. The phloem was sampled from the first-year twigs adjacent to twigs covered with the chambers. For phloem exudation, a bark piece of ca. 2 g was sampled from the cut end of one twig per studied tree. All bark samples were washed with double-distilled water in order to exclude contamination of phloem exudates with xylem sap. Sugars were extracted from phloem of the 1-year old twigs and from the 1-year and 2-year old needles. Proteins were extracted only from the 1-year and 2-year old needles.

### Sugar extraction

A mixture of needles or phloem was homogenized with a small amount of water, liquid nitrogen and ground glass, washed and centrifuged. The precipitate was removed, and the solution filtered under a vacuum (water-jet pump). The phloem sap samples were tenfold diluted with 96 % ethanol, evaporated and dried in a desiccator. The extracts were separated into two fractions by adding petroleum ether and intense shaking. The upper, polar fraction contained dissolved pigments and lipids and the lower, water-soluble fraction included sugars. After removing the polar fraction with a micropipette, the extracts of sugars were diluted by a factor of ten with 96 % ethanol and were subsequently evaporated and dried in a desiccator. These extracts consisted of at least 97 % sugars according to purity analysis using 7890A/7000 Triple Quad GC/MS (Agilent Technology, USA).

### Protein extraction

Needles were mixed with 0.1 M phosphate buffer (Na_2_HPO_4_/NaH_2_PO_4_/H_2_O), liquid nitrogen and ground glass, then homogenized, washed with 0.1 M phosphate buffer and centrifuged. The extraction of homogenate and removal of pigment were done using the same technique as for sugars. Next, chilled (3–4°C) acetone in proportion 3:1 (acetone: homogenate) was added to the protein extracts. After centrifugation of this mixture, the precipitate was washed with distilled water and dried at room temperature.

### Wood sampling

Samples of stem wood including mature phloem, cambium, developing xylem and mature wood were collected from 10 selected pine trees every 10 days over the entire vegetation season. The samples were obtained with a chisel (0.8 cm) around the circumference of the tree stem at ca. 1.3 m height [41]. An average sampling depth of 7 mm ensured complete extraction of xylem developed between sampling intervals. The xylem cells developed between sampling intervals (ten days) were separated with a scalpel under a Stemi 2000 (Zeiss) stereomicroscope. Then, the 10-day xylem cell samples (200–400 μg) were split into two parts. One part was ground and dried for isotope measurements in the xylem, the second part was used to extract stem cellulose and measure isotopes in the cellulose.

### Cellulose extraction

α-cellulose was extracted from wood samples using a multistage procedure including organic solvent extraction, bleaching and separation steps described in detail as method number 6 in table 2 by Boettger et al [47].

### Isotope measurements

Carbon (δ^13^C) and oxygen (δ^18^O) isotopic compositions were measured from (i) bulk needle material; (ii) sucrose and protein fractions extracted from biomass; (iii) cellulose and sucrose in phloem of the first-year twigs and second-year twigs. Each sample was analyzed at least three times.

The isotope values were measured using a Delta S mass spectrometer (Finnigan MAT) coupled with a HEKAtech high temperature (1450°C) pyrolysis reactor. The isotopic ratios were calculated according to the method described by Boettger et al. and Knöller et al. [47,48]. The isotope ratios are reported according to the international VPDB and VSMOW standards. Overall, the precision of the isotope measurements was better than ±0.2‰ for δ^13^C and ±0.3‰ for δ^18^O.

### Statistical analyses

The normal distribution of data was tested. Arithmetic mean values and their standard deviation (SD) were calculated. The conventional Student’s t-test [49] was applied to determine the statistical significance of deviations.

## Authors’ contributions

VV and VO have designed the field experiments, sampling and sample preparation. AI has applied the oscillation model for the interpretation of the results. TB has participated in the design, isotopic measurements and statistical analyses. All authors participated in the final interpretation and drafting of the manuscript. All authors read and approved the final manuscript.
